# Engineered hydrogels for mechanobiology

**DOI:** 10.1038/s43586-022-00179-7

**Published:** 2022-12-15

**Authors:** Ulrich Blache, Eden M. Ford, Byunghang Ha, Laura Rijns, Ovijit Chaudhuri, Patricia Y.W. Dankers, April M. Kloxin, Jess G. Snedeker, Eileen Gentleman

**Affiliations:** 1 Fraunhofer Institute for Cell Therapy and Immunology and Fraunhofer Cluster of Excellence for Immune-Mediated Disease, Leipzig, Germany; 2 Department of Chemical and Biomolecular Engineering, University of Delaware, DE, USA; 3 Department of Mechanical Engineering, Stanford University, CA, USA; 4 Institute for Complex Molecular Systems, Department of Biomedical Engineering, Laboratory of Chemical Biology, Eindhoven University of Technology, PO Box 513, 5600 MB Eindhoven, The Netherlands; 5 Department of Material Science and Engineering, University of Delaware, DE, USA; 6 University Hospital Balgrist and ETH Zurich, Zurich, Switzerland; 7 Centre for Craniofacial and Regenerative Biology, King’s College London, London SE1 9RT, UK

## Abstract

Cells’ local mechanical environment can be as important in guiding cellular responses as many well-characterized biochemical cues. Hydrogels that mimic the native extracellular matrix can provide these mechanical cues to encapsulated cells, allowing for the study of their impact on cellular behaviours. Moreover, by harnessing cellular responses to mechanical cues, hydrogels can be used to create tissues in vitro for regenerative medicine applications and for disease modelling. This Primer outlines the importance and challenges of creating hydrogels that mimic the mechanical and biological properties of the native extracellular matrix. The design of hydrogels for mechanobiology studies is discussed, including appropriate choice of cross-linking chemistry and strategies to tailor hydrogel mechanical cues. Techniques for characterizing hydrogels are explained, highlighting methods used to analyze cell behaviour. Example applications for studying fundamental mechanobiological processes and regenerative therapies are provided, along with a discussion of the limitations of hydrogels as mimetics of the native extracellular matrix. The article ends with an outlook for the field, focusing on emerging technologies that will enable new insights into mechanobiology and its role in tissue homeostasis and disease.

## Introduction

Studies of how soluble cues drive cellular behaviour have given an unprecedented insight into the mechanisms that govern human biology and disease. However, biochemical signals alone do not fully explain many biological phenomena. Instead, diverse processes ranging from cancer metastasis to embryogenesis are governed by both the biochemical cues cells exchange and mechanical signals they generate and receive. Cells respond to extrinsic mechanical cues, such as fluid shear, applied strains and confinement, but they also generate forces to probe the elastic and viscoelastic properties of their local surroundings^1^. A century ago, the first description of how tensile stress in the developing femur favours osteoblastic differentiation was published^2^. Since then, the role of mechanical cues in directing cell migration, maintaining stem cell niches, and in tissue repair have been identified in almost every tissue^3–5^. Even in the immune system, it is now known that leukocytes detect the mechanical properties of cells and tissues, which is essential for their migration and activation, and thus their ability to generate pathogen- and cancer cell-targeting responses^6,7^. Specialized receptors and signaling pathways are activated in response to mechanical cues, which drive changes in gene expression, impacting cell response^8^. In short, cells’ physical environment, including how they sense and generate mechanical cues, play key roles in health and disease.

**Figure 1:**
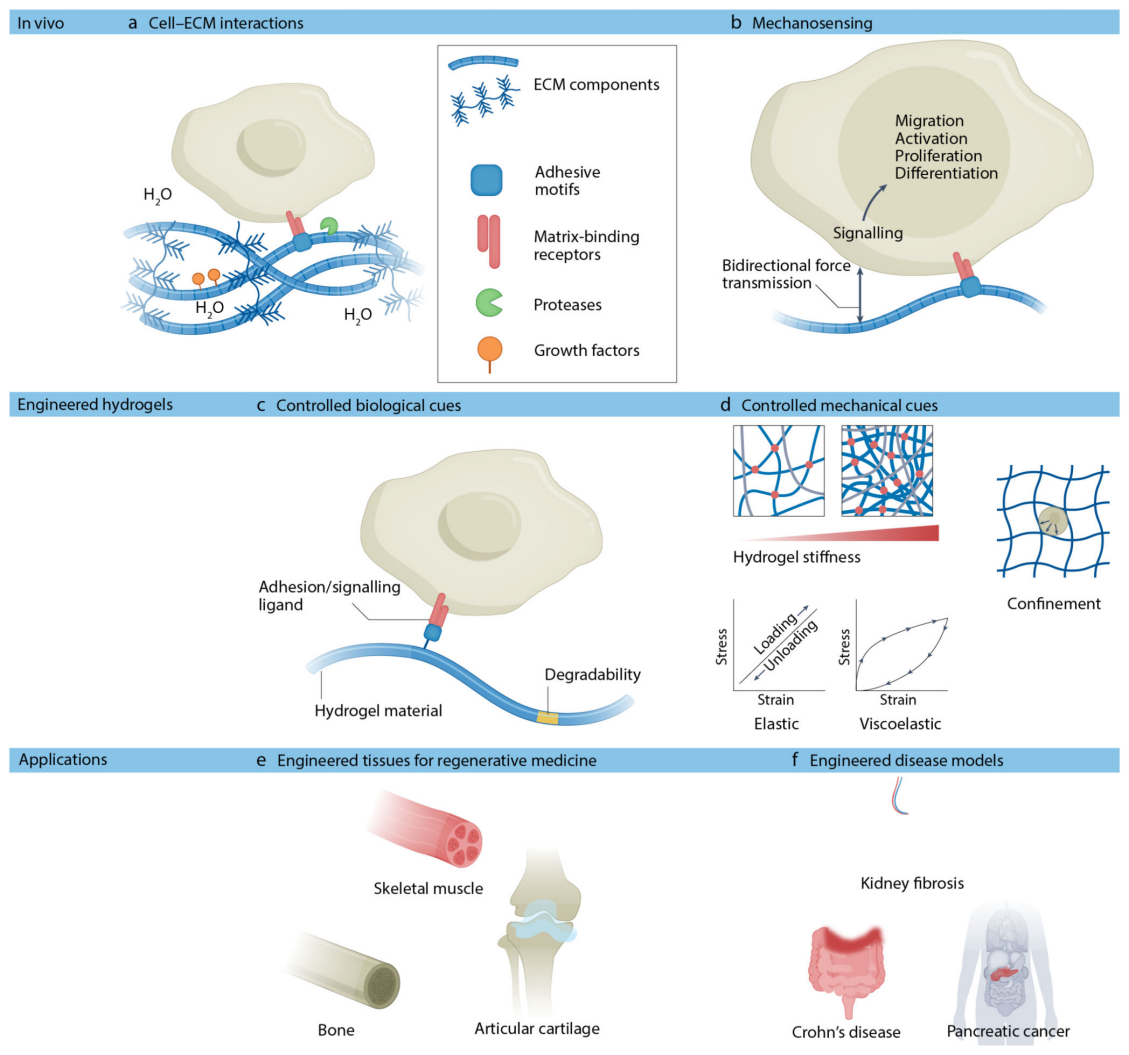
Engineered hydrogels replicate ECM and mechanical cues of native tissues. **a)** In native tissues, cells interact with their surrounding ECM via matrix-binding receptors. **b)** Cells detect mechanical cues in their local environment by applying traction on their surrounding matrix. This process, known as mechanosensing, prompts biochemical signaling, which drives gene transcription and activation of various proteins resulting in cell migration, proliferation and progenitor cell differentiation. **c**) In vivo-like ECM cues can be replicated in hydrogels, often by tethering adhesive motifs copied from the native ECM directly into the hydrogel material. **d**) Many mechanical cues provided by the native environment can be replicated using hydrogels. For example, hydrogel properties can be modulated to mimic the stiffness or viscoelastic properties of a native tissue. Minimally deformable and non-degradable matrices can also be created to confine cells. **e**) Engineered hydrogels have found numerous applications in regenerative medicine and disease modelling. For example, as scaffolds for muscle, bone and cartilage tissue engineering. **f)** Hydrogels have also been applied in disease modelling to provide a local environment to cells and organoids that mimics tissue-specific ECM ligands and local elasticity/viscoelasticity. This approach enables mimicking of pathological changes in the native ECM during fibrotic wound healing and in diseases such as cancer. Abbreviations: ECM, extracellular matrix.

The emergence of the molecular basis of biology drove the development of genetic tools and birthed the field of structural biology. Together, these techniques have provided a mechanistic understanding of biological processes, leading to the development of numerous therapies. With the developing appreciation of mechanobiology, there is similarly a need for new tools and to better understand how physical forces impact cells and tissues, both during homeostasis and in disease. Central to this emerging toolbox are a versatile class of biomaterials called hydrogels. Hydrogels are water-swollen polymer networks that can form under physiological conditions, allowing for the encapsulation of live cells. There are a plethora of tools available to chemically modify hydrogels and control their presentation of biological moieties. Moreover, hydrogels’ mechanical properties can be tuned across a wide range, matching those of many native tissues. As a result, hydrogels can be used as highly controlled mimics of the native extracellular matrix (ECM)^9^ (Figure 1).

Hydrogels have already proved invaluable in shaping a fundamental understanding of how intrinsic mechanical cues drive fate specification^10^. For example, changing the stiffness [G] of a 2D surface can direct human mesenchymal stem cell differentiation (hMSC) in the absence of differential soluble cues^11^. Differentiation was most effective on surfaces that matched the stiffness of the native tissue, suggesting that to create regenerative therapies, biomaterial scaffolds should ideally match the stiffness of the native tissue they aim to replace. Similarly, stem cells isolated from skeletal muscle and grown on substrates that match the stiffness of the native tissue self-renew in vitro and contribute to muscle regeneration in vivo. This contrasts with their regenerative capacity when cultured on rigid tissue culture plastic, where progenitors lose their ability to engraft and mediate repair^12^. Thus, hydrogels that can provide mechanical cues to cells have been harnessed to understand fundamental biological processes and to control cell behaviour for regenerative applications^13^, including in cartilage, bone and muscle repair.

In most tissues, cells do not reside in 2D monolayers, but exist within a complex 3D matrix. Here, hydrogels that can encapsulate live cells are particularly powerful tools. In contrast to 2D, where cells’ ability to spread and apply traction on the underlying surface is unrestricted, in 3D, mechanical cues are more complex as confinement, degradation, stress relaxation [G] and matrix secretion together regulate an intricate interplay between the applied forces, intrinsic mechanical cues and cellular responses. Within covalently cross-linked 3D hydrogels, hMSC are insensitive to changes in stiffness and adopt adipogenic phenotypes. However, when matrix metalloproteinase [G] (MMP)-susceptible peptide sequences are introduced as cross-linkers, cells can remodel their local environment, exert traction on their surrounding matrix, and undergo osteogenesis^14^. Such complex, mechanically regulated responses can have important implications when hydrogels are used to develop regenerative therapies or create human disease models^15,16^. For example, chondrocytes embedded within fast-relaxing ionically cross-linked hydrogels increase in volume and secrete an extensive, cartilage-like matrix. Conversely, confining hydrogels, which do not allow for cell volume expansion, cause encapsulated chondrocytes to upregulate genes associated with cartilage catabolism^17^. Encapsulated pancreatic tumouroids^18^ and kidney organoids [G] ^19^ similarly respond to 3D mechanical cues that mimic fibrotic microenvironments, and intestinal organoids have been shown to modify their surrounding matrix in a process that mirrors pathological matrix remodelling in Crohn’s disease^20^.

This Primer describes hydrogel designs suitable for studying mechanobiology. Methods are explained for generating hydrogels, characterizing their properties and assessing how mechanosensing by encapsulated cells drives biological responses. Examples are given of how hydrogels have been used to identify new and exciting ways that mechanical cues influence cellular responses and how this knowledge has been leveraged for therapeutic applications and to understand human disease. The Primer ends with a discussion of the limitations of current hydrogels technologies and provides a future perspective on how the field may develop in the coming years.

## Experimentation

Creating a hydrogel requires a polymer type to be chosen, and then tailoring its biological and mechanical properties using physical or chemical modifications. This section discusses the general properties of different hydrogel classes, highlights their strengths and limitations (Box 1), and provides examples of systems that display useful biological and mechanical features for studies in mechanobiology.

### Hydrogel material types

**Figure 2:**
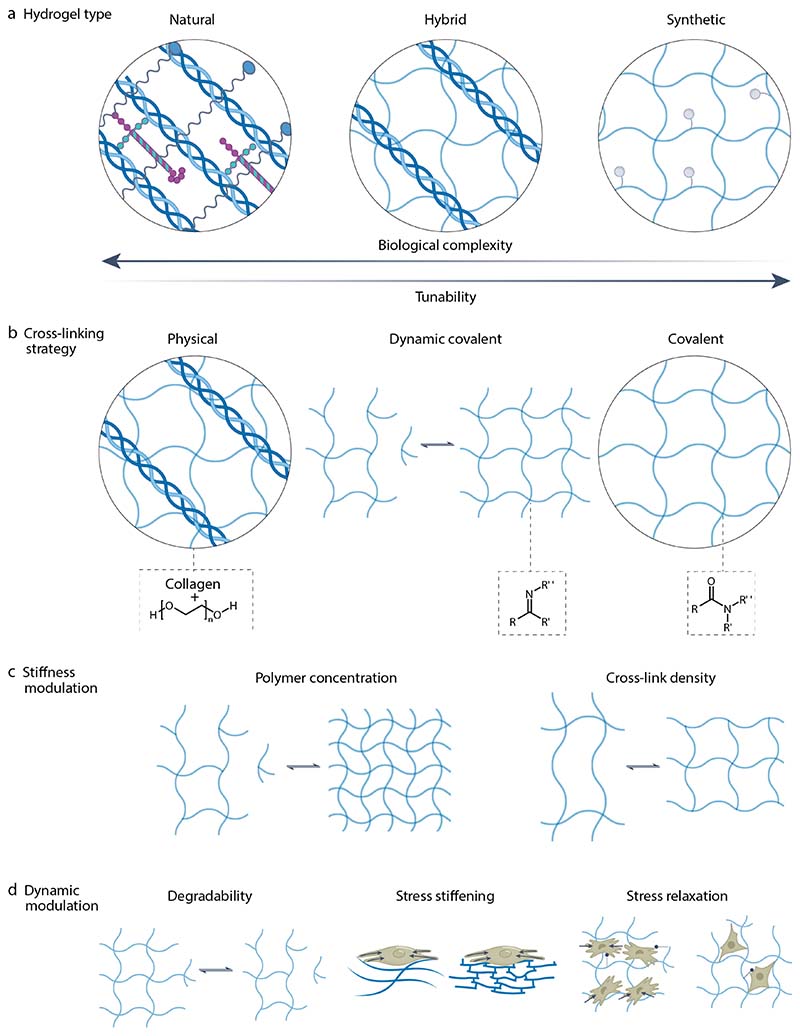
Overview of different types of hydrogels. (A) Hydrogels can be formed from natural or synthetic materials or by making hybrid designs that contain both material types. Natural materials show a high degree of bioactivity, but it is often not possible to orthogonally tune their mechanical or dynamic properties. This contrasts with synthetic materials, which are often highly tunable but are limited in their biological complexity. (B) Hydrogels can be formed using covalent, physical or dynamic-covalent cross-linking strategies. (C) Hydrogels’ mechanical properties can be modulated by varying polymer concentration and/or the cross-linking density. (D) Time-dependent properties including stress stiffening, matrix degradability and stress relaxation can be incorporated into hydrogel designs.

Hydrogels can be made from both natural and synthetic polymers, or using hybrid designs that incorporate both (Figure 2). Hydrogels formed from natural ECM components have long been used to reveal fundamental insights into biology. The main class of hydrogels derived from natural ECM are isolated protein polymers such as collagen type I, gelatin, and fibrin. Alternatively, when a hydrogel is required to closely mimic the biological cues present in a specific tissue, decellularized extracellular matrices can be an effective option^21–23^. Another option is to collect the matrix secreted by cell cultures, as is the case with Matrigel, a laminin-dominated material derived from the Engelbreth-Holm-Swarm mouse sarcoma cell line. Hydrogels made from ECM polymers are well suited for the growth of encapsulated cells as their inherent properties allow interactions with the polymer via cell surface integrins that bind specific ECM motifs. In addition, these hydrogels can be remodelled by enzymatic degradation of the natural polymer by MMPs or plasmin.

Despite these strengths, natural hydrogels often display batch-to-batch variability, which can be challenging for experimental reproducibility. Untangling the role of specific mechanical and biological cues in cell response is difficult due to the complexity of signals naturally derived materials provide to encapsulate cells. Even the growth factor-reduced version of Matrigel contains hundreds of proteins^24^. It is also challenging to modulate the stiffness of natural hydrogels without simultaneously altering their protein density, which can make it difficult to attribute cellular responses to mechanical cues. Although they are touted as a single component, proteins such as collagen are often extracted from animal tissues. Despite being purified, they can still contain other proteins and growth factors, which may impact cellular responses. Additionally, their xenogeneic origin can limit use in translational applications. Another major disadvantage of protein-based natural hydrogels is that their mechanical properties are limited by the protein concentration needed for network formation and by the protein solubility.

To better control their mechanical properties, natural ECM components can be chemically modified or combined with synthetic polymers, creating hybrid hydrogels. For instance, the collagen-derivative gelatin can be stabilized using a methacrylic anhydride modification (GelMA), allowing it to form mechanically stable hydrogels^25^. Similarly, collagen-poly(ethylene glycol) (PEG) hybrid materials have been formed in which the materials’ mechanical properties can be tuned independently of collagen density^26^.

Carbohydrate-based materials have also been widely explored^27^, as they can offer further control over hydrogel properties. For example, hydrogels can be formed from hyaluronic acid, heparin and dextran, which can be made recombinantly, guaranteeing purity. Carbohydrates provide the basis for many commercially available 3D hydrogel systems, such as hyaluronic acid-based HyStem® (Advanced Biosystems), and dextran-based materials such as 3D Life ToGro® (Cellendes) and TrueGel3D™ (MilliporeSigma/Merck KGaA Life Sciences). However, hyaluronic acid, like brown algae-derived alginate, lacks sites for integrin-mediated interactions with cells, limiting its ability to mimic the ECM biologically. For most carbohydrate-based hydrogels, chemical modifications are needed to render them bioactive.

To overcome many limitations of naturally derived hydrogels, researchers have created an array of fully synthetic alternatives. Such chemically synthesized materials are often considered blank slates, as they are free of animal products and their associated confounding biological factors. Examples include hydrogels formed from PEG^28–30^, polyisocyanopeptides^31–33^, and short peptides^34^. Commercially available options for fully synthetic hydrogels include PeptiGels® (Manchester BioGel), which are based on self-assembling peptides, and 3DProSeed®(Ectica Technologies), a PEG-based platform that permits 3D culture, but are provided pre-cast in well plates.

Synthetic hydrogels have been widely used, particularly for mechanobiology studies, because their mechanical properties can be tuned over a wide range and their biological properties can be fully defined. As a result, synthetic hydrogels provide a well-controlled, highly tunable microenvironment for encapsulated cells that can be modulated to mimic key aspects of the native ECM. Despite these advantages, formation of these hydrogels often requires synthetic chemistry approaches to allow for gelation and bioactivity, which can be inaccessible and intimidating to many biological laboratories^35^.

### Incorporating biological cues

Synthetic hydrogels are often made suitable for biological studies by incorporating biological motifs that can be recognized by encapsulated cells, such as peptides that mimic integrin binding domains and enzymatic cleavage sites within native ECM proteins. This is a particularly powerful approach as the incorporation of peptides into synthetic hydrogels can be finely tuned, producing 3D matrices where the density of ECM cues can be altered to match those in the native tissue.

Incorporation of peptides that mimic integrin-binding sequences into synthetic hydrogels is often essential for cell viability^36^. For example, RGD sequence-containing peptides, which mimic the binding sequence in fibronectin and other proteins, is often necessary. Without such binding motifs, encapsulated cells might otherwise undergo anoikis [G]. Other peptide motifs that have been incorporated into synthetic hydrogels include IKVAV and YIGSR, which are derived from the laminin binding sequence, and GFOGER, which when designed to assemble in a triple helix conformation, mimics the binding sequence’s presentation in native collagen^37^. However, even for cells that do not rely on such cell interactions for viability, traction-based mechanosensing [G] often requires adhesive cues.

Another important factor is the incorporation of degradability into hydrogels. Without degradability, most covalently cross-linked networks will confine cells, which can preclude cytoskeletal rearrangements and many forms of mechanosensing. While many hydrogels derived from natural polymers will be degraded by a combination of hydrolysis and enzyme-mediated processes, for hydrogels derived from many non-mammalian sources — cellulose or alginate, for example — and synthetic polymers, degradability must be incorporated into their design. To achieve degradability, hydrogel networks can be cross-linked with peptides that can be cleaved by matrix-degrading enzymes. For example, many PEG networks are cross-linked using peptides containing the sequence GPQG↓IWGC, where the ↓ indicates the enzymatic cleavage site^29^. This sequence is highly susceptible to degradation by mammalian MMPs, including MMPs 1, 2, 7, and 9^38^. Hydrogels have also been designed to be susceptible to degradation by plasmin^39^, and specific enzymes such as A Disintegrin and Metalloprotease 9 (ADAM9)^40^, which is expressed by neural progenitor cells. Encapsulated cells which express these enzymes will, over time, both degrade the matrix locally, allowing for changes in cell morphology and migration, and soften the hydrogel globally.

### Cross-linking strategies

Central to any hydrogel formation strategy is that the polymer material must be cross-linked in the presence of water to create a hydrated network. Cross-linking prevents the hydrophilic polymeric molecules dissolving. Different cross-linking methods exist, including covalent, dynamic-covalent, and physical^41,42^.

Covalently cross-linked hydrogels can be formed with a wide range of mechanical properties, under mild conditions suitable for cell encapsulation. Covalent cross-linking can be achieved using photo-initiated or radical-initiated strategies, such as Irgacure 2959, lithium phenyl-2,4,6-trimethylbenzoylphosphinate (LAP), or N,N,N’N’-tetramethylene-diamine (TEMED) combined with a peroxydisulfate^43^; functional groups, including amine-carboxylic acid, thiol-ene, copper-catalyzed azide-alkyne cycloadditions^44,45^; and reactions catalyzed by enzymes, for example Ca^2+^-dependent transglutaminases^46,47^. The irreversibility of covalent cross-links means that covalently cross-linked hydrogels can often lack the time-dependent mechanical behaviours inherent to native tissues, like stress relaxation. To overcome this problem, hydrogels can be designed using dynamic-covalent cross-linking (DCC) strategies, whereby covalent bonds dissociate and re-associate. This enables the network to accommodate cellular processes, such as cytoskeletal re-arrangements and migration. Well-known examples include Diels Alder, boronate ester, thiol-disulfide, oxime and imine cross-links^42^. DCC hydrogels can be formed with a wide range of stiffnesses and can incorporate viscoelastic properties^48^. However, as bulk stability is determined by the kinetics of the forward and reverse reactions, they tend to swell over time and become unstable^42^. Because of this property, DCC hydrogels are often challenging to tune, as subtle changes in the kinetics can have a profound impact on the hydrogels’ mechanical properties and stability.

Physically cross-linked hydrogels, sometimes referred to as supramolecular hydrogels, are networks that rely on less stable physical associations, such as ionic interactions, protein interactions (antibody—antigen pairs), hydrogen bonding, π–π interactions, and the hydrophobic effect^49^. These physical interactions lend the hydrogels dynamic properties, such as stress relaxation, while still controlling mechanical stiffness. One of the most common physically cross-linked hydrogels for biological applications relies on alternating blocks of sugars within alginate interacting with divalent cations. Well-known monomeric building blocks also include DNA^50^, peptide amphiphiles^51^, cytosine^52^, ureido-pyrimidinone^53,54^, and the benzene-1,3,5-tricarboxamide motif^55,56^. Disadvantages of physically cross-linked networks include the potential presence of hydrophobic and hydrophilic regions within the hydrogel. The dynamic nature of the systems also means that they can be unstable. Moreover, the dynamic nature of alginate hydrogels, whose stiffness is often altered by changing the concentration of the divalent cation Ca^2+^, can potentially impact cellular processes, particularly in cells that are highly susceptible to calcium concentrations.

It is also possible to combine cross-linking strategies in the same hydrogel by creating interpenetrating networks (IPNs) composed of two entangled networks, often cross-linked using different approaches. For example, IPNs can be formed by combining a covalently cross-linked primary network with a secondary network that relies on supramolecular interactions^57,58^. IPNs have the potential to provide the stability and strength of covalently cross-linked hydrogels with the of ease of remodelling inherent to dynamic networks.

### Hydrogel stiffness

The term stiffness is often used interchangeably with elasticity or compliance^59^. The relative stiffness of an elastic material can be quantified by its elastic modulus [G], or the tangent of the material stress-strain curve for a given amount of deformation, which is an intrinsic material property that is independent of the material’s size. If the material stress-strain curve is linear, the elastic modulus can be referred to as the Young’s modulus. A hydrogels’ stiffness can often be tuned over a wide range depending on the chemistry and base material. Hydrogels based on natural polymers, if not chemically modified, will generally have lower bulk stiffnesses — maximum elastic modulus of a few hundred Pa — while hydrogels based on synthetic polymers offer a much wider stiffness window, reaching ˜10s-100s kPa. Consequently, synthetic hydrogels — which can be formed to match the mechanical stiffness of tissues ranging from brain (hundreds of Pa), to abdominal organs (intestine, ˜1kPa), and connective tissues such as cartilage (˜MPa)^60^ — are often preferable for mechanobiology studies due to their tunability and more controllable mechanical properties.

Hydrogel stiffness is often modulated by altering either the polymer concentration, the density of cross-links, or both. For naturally derived materials like collagen, stiffness can be altered by changing polymer concentration. However, this impacts the density of ligands presented to cells. Methods have been described to alter collagen hydrogel stiffness without altering fiber architecture or polymer concentration^61^, but such approaches only offer a narrow range of stiffnesses. In many synthetic systems, stiffness can be controlled independently of ligand density. Changing stiffness can also impact other hydrogel properties, such as mesh size [G] or pore size. Mesh size refers to the distance between molecular cross-links and influences the ease with which molecules, such as nutrients, diffuse within a hydrogel^62^. Generally, mesh size is coupled to stiffness, as increasing hydrogel stiffness usually results in smaller mesh sizes and vice versa.

### Hydrogels with dynamic properties

Stiffness is not the only property that cells respond to; they can also change their behaviour in response to a material’s viscoelasticity [G], as characterized by stress relaxation behaviors^63^ and nonlinear elasticity, such as strain stiffening^31^. Stress relaxation is often reported as the relaxation half-time. This is the time required for a material to relax to half of its initial stress, which can be on the order of seconds to minutes for many soft tissues^64^. In 3D, viscoelastic responses can allow encapsulated cells to adopt spread morphologies and remodel their surroundings through matrix secretion, without a requirement for degradation.

Modulation of hydrogel viscoelastic properties can be achieved using different strategies. Natural ECM-based matrices, including those formed from collagen, fibrin, or Matrigel, typically exhibit substantial stress relaxation^65,66^, but offer limited independent tunability. Viscoelastic responses can be reduced by adding covalent cross-links, but this can impact stiffness. For other hydrogels, viscoelasticity must be incorporated as part of their chemistry. For example, strain stiffening hydrogels have been created using fully synthetic polyisocyanopeptides^31^, and stress relaxation in alginate hydrogels can be modulated by altering the molecular weight of the alginate molecules and the ionic cross-linking density. High molecular weight alginates will relax more slowly than low molecular weight alginates, allowing modulation of viscoelastic properties while holding the hydrogels’ stiffness constant^63^. In PEG hydrogels, the rate of stress relaxation can be modulated by varying the ratio of dynamic covalent cross-links with differing unbinding kinetics^67^, or by using a combination of covalent cross-linking and a triple hydrogel bonding interaction^52^. The properties of hydrogels that fully rely on supramolecular interactions can also be modulated. For example, hydrogels whose cross-linking relies on hydrogen bonding interactions can be regulated either by changing the packing and overall hydrogen bonding strength^68^, or by using different supramolecular monomers, which influences cross-linking and nanofiber formation^54^. In this way, supramolecular hydrogels can be rendered dynamic at the mesoscopic level and also at the molecular level by inducing monomer association-dissociation to enhance supramolecular motion. This results in stress relaxation behaviour.

Modulation of hydrogel physical properties can be introduced to allow for real-time, in situ and use-triggered modulation of cells’ microenvironment. Using light- or enzymatically sensitive groups, mechanical properties can be modulated and adhesive groups exposed or released. Strategies to achieve this often rely on photo-sensitive groups, such as 3-(4,5-dimethoxy-2-nitrophenyl)-2-butyl esters^69^ or nitrobenzyl^70^, which can be cleaved by a high energy light source and conversely, methacrylate^14^ or thiol-ene pairs^71^, which can trigger local photo-sensitive cross-linking. Using these strategies, light-responsive hydrogels that controllably soften and stiffen^72^, and switch between being cell adhesive and non-adhesive^69^ have been created.

## Results

As many cell types respond to mechanical cues, it is important to understand hydrogels’ properties — including swelling behaviour, architecture, elastic and viscoelastic properties — to attribute biological responses to physical cues. Standard biological techniques for analyzing cell behaviour on 2D surfaces are not always applicable in 3D. As a result, techniques need to be adapted to make them suitable to study cells within hydrogels. This section describes approaches to characterize the bulk properties of hydrogels, quantify cells’ mechanical interactions with their surrounding matrix, and assess cellular phenotypes in response to mechanical cues.

### Swelling and mesh size

**Figure 3:**
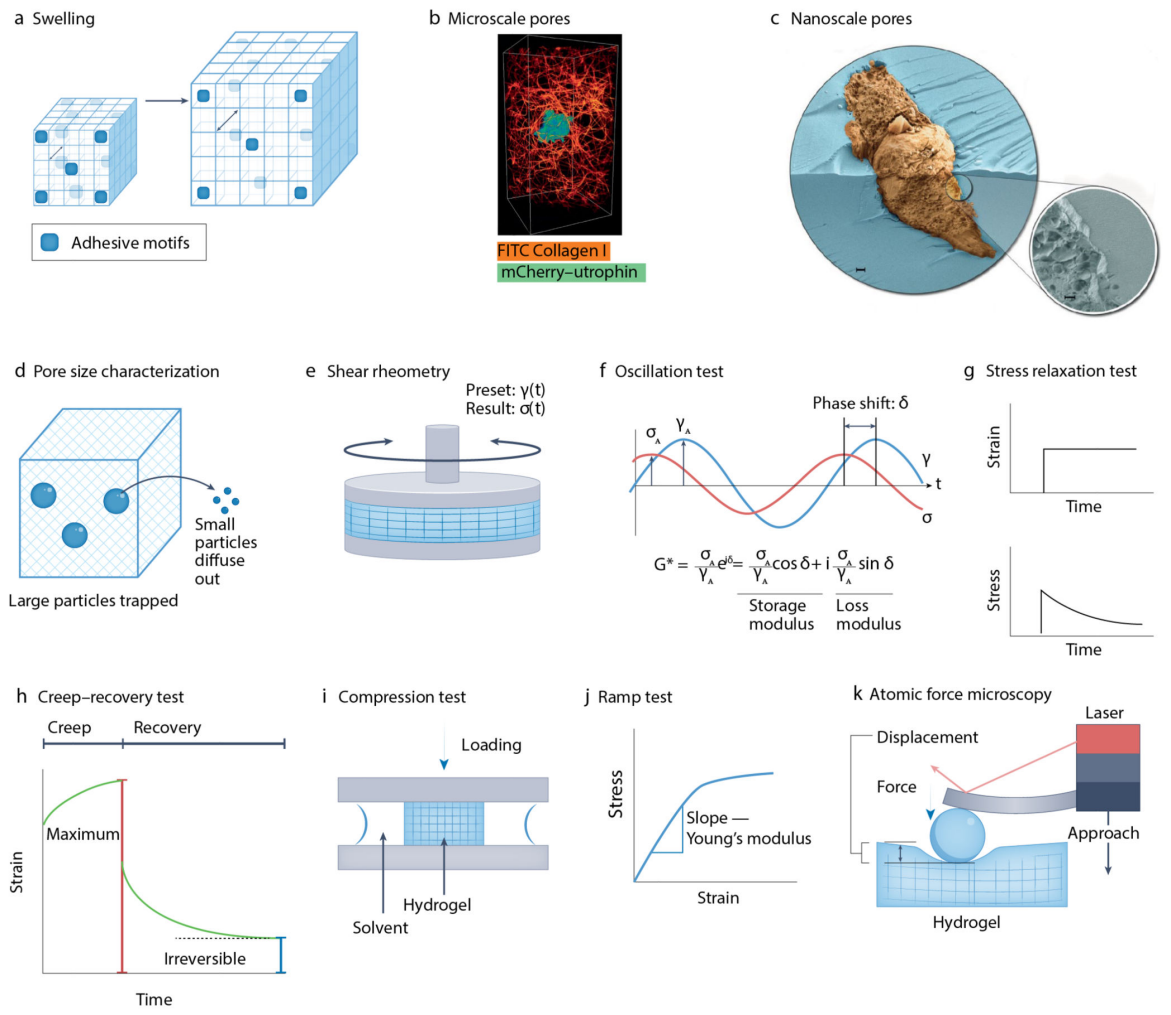
Measuring physical properties of hydrogels. **a)** Hydrogel swelling and associated changes in mesh size and ligand density. **b)** Fluorescence light-sheet micrograph showing a neutrophil-like human HL-60 cell expressing mCherry-utrophin in a fluorescently labelled collagen matrix. **c)** False-coloured cryogenic-scanning electron microscopy (SEM) micrograph showing the 3D interface between a fully hydrated hydrogel (blue) and an encapsulated cell (brown). Scale bar, 1 µm. **d)** Mesh size characterization using beads with known sizes. Small beads will diffuse within the hydrogel and be detected within the surrounding fluid whereas large beads will remain entrapped, providing an indication of mesh size. **e**) Operation of rotational rheometer. **f**) Example of a sinusoidal stress (σ)-strain (γ) curve and how storage and loss moduli are defined using the peak amplitudes (σA,γA) and phase shift (δ). **g**) General features of material response in a stress relaxation test. **h**) General features of material response in a creep-recovery test. **i**) Operation of a compression testing device. **j)** General behaviour of a hydrogel during a ramp test. **k)** Operation of an atomic force microscope^199^ and general features of a hydrogel response during a cycle of approach and retraction of the atomic force microscope cantilever. Part b reprinted with permission from ref^91^. Part c adapted with permission from ref^199^. Part h reprinted with permission from ref^82^.

Because of their inherent hydrophilicity, hydrogels often swell when placed in cell culture media, due to polymer, solute, and water interactions. Swelling is typically measured by taking the ratio of the of the hydrated hydrogel mass to its dry mass. It is important to understand hydrogel swelling because, in addition to stretching polymer chains and altering hydrogel mechanical properties, swelling can also change the density of biochemical cues within the hydrogel (Fig. 3a). Consequently, differential swelling between hydrogels that have, for example, different stiffnesses, can alter the density of tethered ligands, making it difficult to determine if a cellular response is attributable to the change in stiffness or the change in ligand density.

Another important factor is hydrogel mesh or pore size, which describes the distance between cross-links in the network and can impact solute diffusivity and mechanically confine cells. While natural hydrogels formed from collagen or fibrin can have mesh sizes on the order of microns, which can allow cells to migrate in the absence of degradation or the breaking of cross-links, many synthetic hydrogels have mesh sizes on the nanometer scale. Such small mesh sizes are due to high polymer concentrations and because the polymers chains do not typically bundle into thick fibers, similar to native ECM molecules like collagen. This confines cells, making it nearly impossible for them to migrate or change their morphology without altering the hydrogel.

Hydrogel architecture can be visualized using microscopy and characterized by quantitative image analysis to provide an indication of mesh size. For example, optical microscopy — such as confocal fluorescence, reflectance or second harmonic generation — is suitable for visualizing native fibrous structures in protein hydrogels like collagen fibers^73^ (Fig. 3b). However, the resolution of optical techniques is on the order of hundreds of nanometers at best, meaning they are not capable of imaging nanoscale structures. For higher resolution imaging, scanning electron microscopy (SEM) can be used, but SEM preparation can alter the mesh. Cryo-SEM can reduce these artefacts and better preserve the native hydrogel structure^74,75^ (Fig. 3c), however, it remains challenging to visualize a native mesh. Mesh sizes can be also determined using diffusivity measurements and theoretical models^62,76^. Online tools have been developed to estimate mesh size based on hydrogel characteristics^77^. Diffusivities of macromolecules — such as proteins, dextrans, and DNA — of known size in swollen hydrogels can be measured in situ using fluorescence recovery after photobleaching (FRAP)^78,79^ or gel electrophoresis^80^. Alternatively, a simple and model-independent way of estimating mesh size is to embed pegylated gold nanospheres or polymer beads with varying sizes in the hydrogel, then determine the minimum size where the nanoparticles become trapped^81^ (Fig. 3d).

### Mechanical properties

The elastic or viscoelastic properties of hydrogels can be measured using shear rheometry or compression testing. During shear rheometry, a hydrogel is sandwiched between two flat plates. Shear stresses (loading) or shear strains (deformation) are applied by rotating one plate relative to the other, and the resulting strain or stress measured (Fig. 3e). Adhesion of the hydrogel to the plates is critical to avoid slippage and can be facilitated by forming the hydrogel in the plates or coating the plates with an agent that promotes adhesion. During a dynamic test, an oscillatory stress or strain is applied to the sample. For a purely elastic material, stress and strain will be perfectly in-phase, with the amplitude of the response indicating the elasticity. By contrast, the response will be completely out-of-phase for a purely viscous fluid. Viscoelastic materials have a response between these two extremes. The in-phase component is described as the storage modulus, a measure of elasticity, while the out-of-phase component is defined as the loss modulus, a measure of viscous energy dissipation (Fig. 3f).

In a stress relaxation test, a step strain is applied to the material and the resulting stress is measured over time (Fig. 3g). Stress in this geometry corresponds to the resistance to deformation and viscoelastic hydrogels undergo a reduction in stress over time. Relaxation can be described by the relaxation half time, a model-dependent lifetime defined as the time required for the stress to reduce to half its initial value^63^. Creep-recovery tests can also be used to characterize a hydrogel’s plasticity, or the extent to which it exhibits irreversible deformation following loading. Following loading (creep [G]) and unloading (recovery), hydrogels that are mechanically plastic can be described using the ratio of irreversible strain to maximum strain^82^ (Fig. 3h).

Compression testing involves application of normal stresses or strains to a sample between two parallel plates in a mechanical testing machine, typically with the hydrogel being unconfined laterally (Fig. 3i). By ramping up the strain, the hydrogel’s modulus can be obtained as the slope of the resulting stress-strain curve at small strains (Fig. 3j). Stress relaxation tests can similarly be performed by holding the strain constant and measuring the stress over time. Compression tests conducted on hydrogels can be complex, as compressing the material can change its volume and shape. As a result, stress relaxation due to water movement in the hydrogel, known as a poroelastic effect, must be considered^83^. Of note, the mechanical properties of hydrogels are often described using a single measure of modulus or relaxation half time, but these simple measures do not capture the full mechanical behaviour of many complex hydrogel materials. For example, hydrogels formed from naturally derived materials such as collagen and fibrin^84^ can also display non-linear properties and their behaviour can be dependent on the magnitude or rate of the input stress or strain.

For some applications, probing the hydrogel’s properties at a smaller scale — such as the scale of a single cell or group of cells — is important. This is particularly crucial for composite hydrogels, whose mechanical properties will often be heterogenous. Both confocal microscopy and SEM have been used to visualize hydrogel heterogeneity by identifying variations in density or phase separation^85^. Atomic Force Microscopy (AFM) applied in force spectroscopy mode has also been applied to measure hydrogel mechanical properties at a small scale and detect heterogeneity. AFM involves local indentation of the hydrogel surface using a soft cantilever modified with a small bead. As the cantilever bends, its deflection is detected using a laser beam^86^ (Fig. 3k).

### Analysis of cells in hydrogels

#### In situ imaging

**Figure 4:**
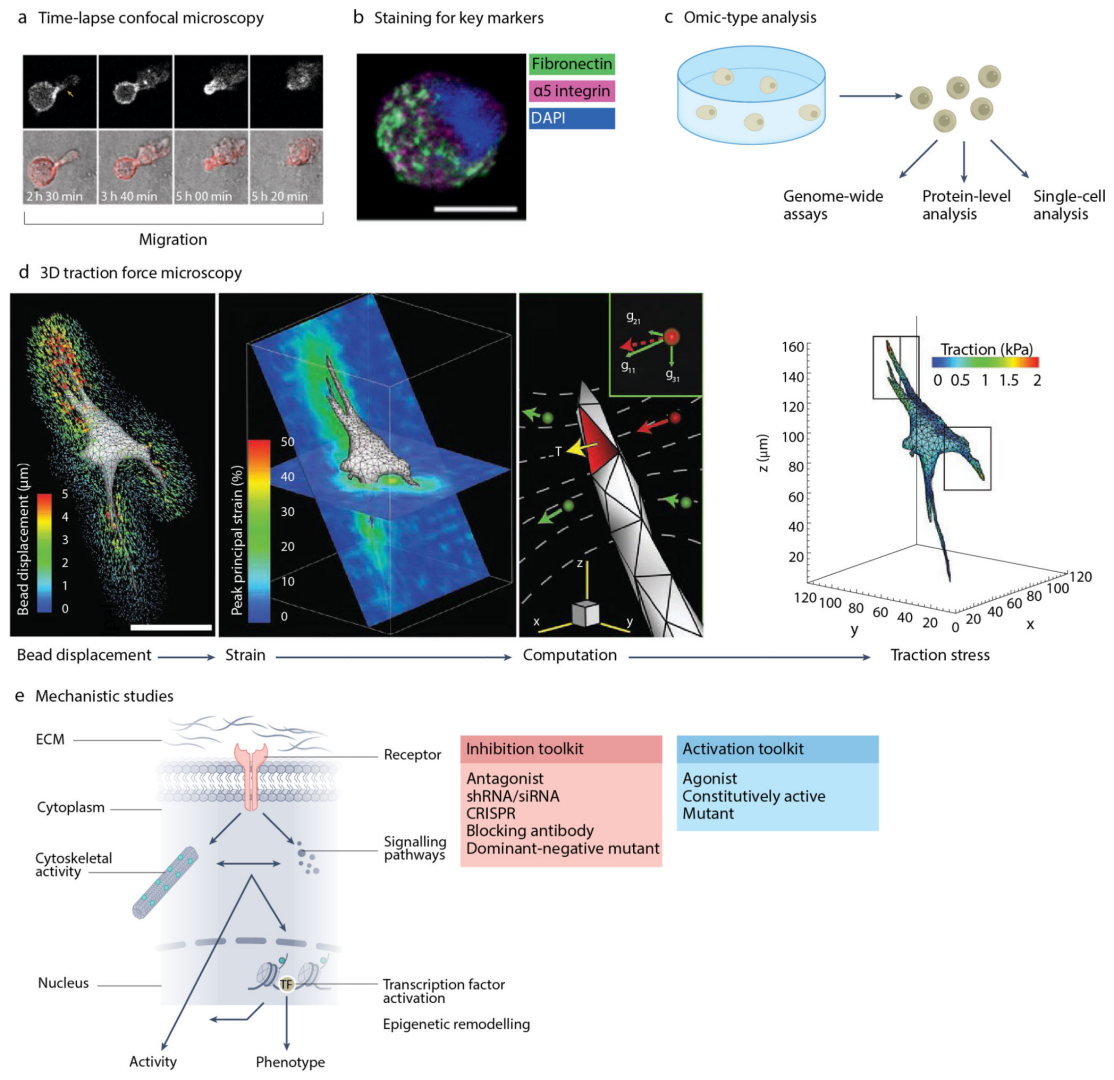
Analysis of cells in hydrogels. **a)** Time series of images of a cancer cell migrating in a hydrogel using time-lapse confocal microscopy. Top row shows images of RFP-LifeAct whereas bottom row combines brightfield and actin imaging. **b**, Human mesenchymal stem cell (hMSC) encapsulated in a hydrogel. Immunostaining for fibronectin, α5 integrin, and DAPI. Scale bar = 10µm. **c)** Omics-type analyses can be used to assess gene expression, chromatin accessibility, protein levels and phosphorylation, and single-cell characteristics. **d**) Pipeline for determining traction forces generated by cells within a hydrogel from bead displacements. Scale bar = 50µm. **e)** Mechanotransduction pathways typically involve activation of a membrane receptor, which causes subsequent cytoskeletal activity and signaling pathway activation, impacting epigenetic remodelling and transcription factor activation, together regulating cell activity and phenotype. Specific molecules can be inhibited or activated to assess their role in cell behaviour. Part a is adapted from ref^81^, CC BY 4.0 (https://creativecommons.org/licenses/by/4.0/). Part b is adapted from ref^109^, CC BY 4.0 (https://creativecommons.org/licenses/by/4.0/). Part c is adapted from ref^138^. Part d is adapted from ref^103^, Springer Nature Limited.

Following encapsulation, various techniques can be performed to analyze cells within hydrogels (Figure 4). An important first step is to assess cell viability, as some cross-linking chemistries and gelation conditions have the potential to be cytotoxic. Simple live/dead staining using fluorescent dyes is often a robust means to quantitatively assess the percentage of live cells. For studies of dynamic cellular processes, including proliferation, morphological changes, and migration, live imaging can be used (Fig. 4a). Most hydrogels used for 3D culture are transparent and amenable to imaging. As with 2D studies, any structure that can be fluorescently labelled or expressed with a fluorescent protein tag can be studied in 3D. Indeed, many studies follow the dynamics of the membrane, actin cytoskeleton, microtubules, and nucleus using commercially available fluorescent molecules to tag these structures.

Confocal microscopy using objectives with long working distances that allow imaging deep into the hydrogel are often applied. Imaging sufficiently deep into the hydrogel is important, as cells can feel the stiffness of an underlying substrate if close to the surface^87^. For 2D polyacrylamide gels, this distance is in the range of 10–20 µm; however, this effect may be material dependent and multiple cells acting in concert can feel an underlying substrate through a thicker gel^88^. Using fluorescence microscopy to image cells within 3D hydrogels with high spatiotemporal resolution is a challenge. High resolution objectives with high numerical apertures typically have low working distances (<200 µm). Further, total internal reflection microscopy (TIRF) and super-resolution imaging techniques that rely on TIRF cannot be used to image into a hydrogel, since TIRF only works within a few hundred nanometers of a microscope slide. Therefore, expansion microscopy, which physically expands the hydrogel and encapsulated cells^89,90^, as well as light sheet and lattice light sheet microscopy^91^ hold potential for improving the spatiotemporal resolution of cells imaged within 3D hydrogels.

#### Staining and molecular analyses

Many standard techniques to analyze cell behaviour in 2D cultures can be adapted to 3D hydrogels. For example, hydrogels can be fixed, cryo-sectioned, and stained using immunostaining approaches to identify the presence or localization of a protein of interest (Fig. 4b). Emerging methods using multiplexed immunostaining^92,93^ are likely to prove invaluable as the field develops. Similarly, in situ hybridization can be performed to visualize expression patterns of specific genes. Standard histochemical techniques, such as H&E staining, can also be applied, but their success will be dependent on hydrogel chemistry as dehydration procedures can destroy or deform some hydrogels, such as those formed from PEG^94^ and hyaluronic acid. Sectioning is often required to effectively stain samples, as the mesh size of many hydrogels can restrict antibody diffusion. Hydrogels can also be digested and processed for biochemical assays, such as to quantify the deposition of specific matrix proteins. The ideal process for staining cells within a hydrogel or digesting it for biochemical analyses is often dependent on the hydrogel material. For example, synthetic hydrogels, which tend to have smaller mesh sizes than those formed from naturally derived materials, often require extended staining times. Digesting hydrogels for biochemical analysis also often requires incubation with specific enzymes to disrupt the network, for instance hyaluronidase to digest hyaluronic acid-based hydrogels. Alternatively, synthetic hydrogels have been created with digestibility by the microbial peptide Sortase A engineered into their design^95^, specifically for this purpose.

In addition to biochemical and imaging techniques, cells in 3D hydrogels can also be processed for molecular analyses. For example, cells can be extracted from PEG-based hydrogels for single cell analysis of cell surface markers using flow cytometry^18^. Numerous -omics based techniques can also be performed, both at the single cell and bulk level, dependent on the ease with which the hydrogel chemistry allows intact cells to be removed (Fig. 4c). For example, RNA-seq can be performed on bulk samples to assess gene expression^96^, and ATAC-seq to assess chromatin accessibility^97,98^, often after homogenizing the whole gel. Conversely, spatial transcriptomics and single-cell RNA-seq can in principle be performed on sections and cells extracted from hydrogels, though these techniques have not yet been used extensively with 3D hydrogel cultures. Analyses of encapsulated cells at the protein level can include phosphoprotein arrays that identify the phosphorylation state of signaling proteins; cytokine arrays that identify secreted cytokines^99,100^; and quantitative mass spectrometry to identify protein levels and modifications more broadly^101,102^. Two major challenges with these analyses are the low numbers of cells typically encapsulated within hydrogels, and the difficulty of extracting cells from some matrices.

#### Assessing cell-hydrogel interactions and underlying mechanisms

In complex 3D environments, it is important to understand how cells modulate and respond to mechanical and biological cues as basic processes such as cell division and migration often require cells to push or pull on their surroundings. To quantify local cell-mediated deformations, it is possible to track the displacement of fluorescent beads embedded within the hydrogel. Techniques have been developed to convert bead movement into 3D traction strain fields in both elastic^103^ and viscoelastic hydrogels^104^ using either a linear elastic continuum mechanics framework or by incorporating viscous materials properties (Fig. 4d). Another potential strategy to quantify cell-generated forces is to use FRET-based sensors, which convert the mechanical tension within a molecule into a fluorescence signal. FRET-based sensors can be created with exquisite sensitivity and have proven to be powerful tools for determining forces exerted on individual molecules within a cell^105^, and applied by cells on 2D surfaces^106^. Nevertheless, while these sensors offer great promise for studying mechanosensing in 3D, thus far, FRET has only been applied to monitor MMP activity within PEG-based hydrogels^107^. This may be due to the challenges of imaging at high spatiotemporal resolution in 3D, however, emerging imaging modalities may offer the possibility of such measurements in the future.

In addition to mechanically remodelling hydrogels, cells can also degrade hydrogels that contain proteolytically susceptible motifs. Recent studies have highlighted the role of matrix deposition in cell-hydrogel interactions. In some contexts, cells secrete their own pericellular matrix^108–111^, which they subsequently interact with. To monitor changes in hydrogel mechanics locally due to remodelling, AFM and microrheology [G] techniques have been applied^20^. Passive microrheology involves embedding small fiducial beads in a hydrogel and then tracking their thermal fluctuations. This technique can be used to measure local gel-sol transitions resulting from protease-dependent hydrogel degradation^112^. Active microrheology involves tracking particle motion driven by an optical trap^113–115^ or magnetic field gradient^116^. In active microrheology, microparticles deform the material locally to probe its mechanical response. However, particle motion can be minimal in stiff hydrogels, limiting the applicability of these techniques to very soft systems.

Alongside techniques that allow researchers to understand how cells modulate their local environment, it is often important to assess cells’ downstream molecular responses to changing mechanical cues (Fig. 4e). Integrins at the cell surface allow cells to exert forces directly on the surrounding hydrogel, while mechanosensitive ion channels respond to membrane tension and their activation might be impacted by cell-hydrogel interactions. Binding of integrins initiates the formation of adhesion complexes, involving mechanosensitive proteins, such as vinculin, talin and focal adhesion kinase, plus activation of downstream signaling pathways, such as Rho GTPase-mediated actomyosin contractility^105^. These signaling pathways and mechanical connections to the lamin cortex of the nucleus^117^ can regulate activation of transcription factors, transcriptional regulators — for example YAP and changes in the epigenome, that together control cellular responses. To analyze cellular responses mechanistically, hydrogel cultures are often amenable to small molecule, protein and molecular manipulations, such as pharmacological inhibition, blocking antibodies, and siRNA-mediated or shRNA-mediated knockdown. Similarly, novel mechanistic players can be identified using transcriptomic, epigenetic or proteomic approaches and CRISPR/Cas9 editing applied to knockout or overexpress specific genes that might play a role in cellular responses to changes in their local environment.

## Applications

Cells sense, respond to, and reorganize their extracellular environments, relying on processes that range in time scales from seconds to weeks^118^. Hydrogels can be designed to direct these dynamic cellular processes for various applications. This section highlights examples where hydrogels have been applied to understand how mechanical cues impact cell behaviours at a fundamental level and how those findings have been harnessed in tissue engineering [G] and disease modelling. The initial focus is on stem cell differentiation as an archetypal example of mechanotransduction [G], before the discussion moves to how hydrogel properties have been harnessed in regenerative medicine, organoid bioengineering, and the emerging field of immunotherapies.

### Stem cell differentiation

**Figure 5:**
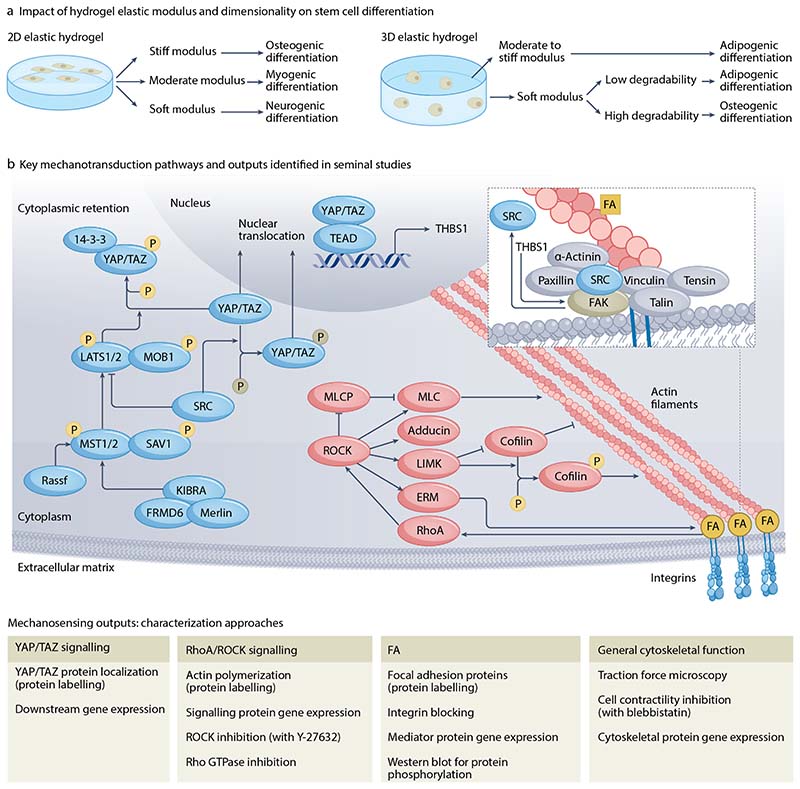
Hydrogels for directing stem cell fate by engaging mechanotransduction pathways. **a)** Hydrogel modulus and dimensionality impact cellular mechanotransduction. Their impact on promoting human mesenchymal stem cell (hMSC) differentiation is highlighted. Hydrogels can be designed with specific mechanical properties to promote stem cell fate specification. **b)** Seminal studies with hMSCs, amongst other cell types, have elucidated that mechanotransduction is mediated, in part, by YAP/TAZ and RhoA/ROCK signaling, focal adhesion formation and more generally the cytoskeleton. Key pathways are noted along with approaches for characterizing them. 14-3-3, tyrosine 3-monooxygenase/tryptophan 5-monooxygenase activation protein; ERM, ezrin/radixin/moesin protein family; FA, focal adhesion complex; FAK, focal adhesion kinase; FRMD6, FERM (4.1-ezrin-radixin-moesin) domain-containing protein 6; KIBRA, kidney and brain expressed protein; LATS1/2, serine/threonine-protein kinase 1/2; LIMK, LIM domain kinase; MLC, myosin light chain; MLCP, myosin light chain phosphatase; MOB1, monopolar spindle-one-binder 1; MST1/2, macrophage stimulatory protein 1/2; P, phosphate group (indicates phosphorylation and dephosphorylation); Rassf, Ras association domain-containing family; RhoA, Ras homolog family member A; ROCK, Rho-associated protein kinase; SAV1, protein salvador homolog 1; SRC, proto-oncogene tyrosine-protein kinase Src; TAZ, transcriptional co-activator with a PDZ-binding motif; TEAD, transcriptional enhancer factor TEF; THBS1, thrombospondin 1 (gene), YAP, yes-associated protein.

Hydrogels have enabled fundamental discoveries, particularly in stem cell fate specification. For example, seminal studies showed that hMSC cultured on 2D polyacrylamide hydrogels, created to match the stiffnesses of brain, muscle, and the developing osteon, upregulated expression of genes implicated in neurogenesis, myogenesis, and osteogenesis, respectively^11^ (Figure 5A). These same 2D hydrogels were later used to identify the role of Hippo pathway co-transcriptional regulators YAP and TAZ in hMSC fate specification^119,120^. Together, these studies exploited mechanical cues received from 2D hydrogel surfaces to highlight how specific signaling pathways and cytoskeletal machinery — including the RhoA/ROCK pathway and focal adhesion complexes — drive fundamental cellular processes (Figure 5B).

However, as most tissues provide 3D environments for cells, there is a need to study mechanotransduction in three dimensions. Differences in stem cell fate were observed when cells were seeded on top of (osteogenesis)^11,121^ versus encapsulated within (adipogenesis) hydrogels of the same stiffness^14^. Stiffness-sensing in 3D is more complex than on 2D surfaces. For example, when encapsulated within covalently cross-linked RGD-functionalized hyaluronic acid hydrogels, hMSCs’ ability to apply traction on their surroundings depends on degradation^14^. By controlling hydrogel degradability and the cells’ ability to spread and generate cytoskeletal tension, fate specification could be altered. Further studies have shown that within physically cross-linked alginate hydrogels, matrix elasticity directs hMSC fate in part through clustering of adhesive ligands^122^.

In comparison, hMSC cultured within stress-relaxing hydrogels show remarkably different behaviour compared to those within purely elastic matrices that do not degrade. For example, within fast relaxing hydrogels, cells have increased cell and nuclear volumes, changes in morphology, and YAP/TAZ localize to cell nuclei. Faster stress relaxation times have also been shown to promote proliferation, spreading, and osteogenesis in murine MSC^63^. Stress stiffening [G] can be tuned to modulate hMSC fate as hydrogels that stiffen at higher stresses promote osteogenic differentiation, in a process that is dependent on microtubule dynamics^31^. Such processes can be further complicated, as hMSC encapsulated within hydrogels do not solely detect mechanical and biological cues provided by the hydrogel^123^. Instead, nascent protein deposition and remodeling rapidly supplants interactions between hMSC and integrin-binding motifs presented by the original engineered matrix. These cell-secreted proteins have been shown to be critical for cell spreading and cell-matrix interactions, as they impact YAP/TAZ signaling and stem cell fate^108–110^.

**Figure 6:**
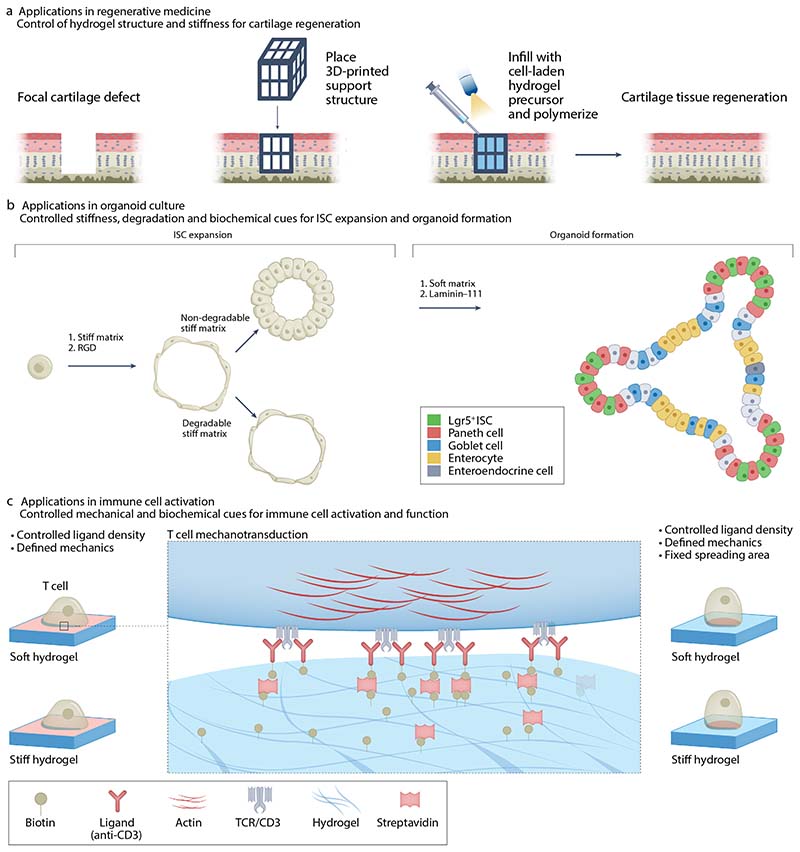
Engineering hydrogel properties for applications in regenerative medicine, organoid groth and and immune cell activation. **a)** Use of a hydrogel-containing hybrid scaffold engineered to mechanically support the surrounding tissue and promote tissue growth for cartilage regeneration. **b)** Stages of intestinal stem cell (ISC) expansion and lumen development, followed by organoid formation. Key matrix requirements for each stage are outlined. **c)** Key properties associated with T-cell mechanosensing and activation. Hydrogels (blue) can be designed to direct immune cell activation and function for a range of purposes from modulating the foreign body response to the production of cell therapies. The approach depicted here controls spreading are by presenting ligands (pink) at the surface of the hydrogel either uniformly (left, an uncontrolled spreading area) or in a controlled manner (right, fixed spreading area). TCR, T cell receptor. Part a reprinted with permission from ref^125^. Part b reprinted from ref^135^. Part c adapted with permission from ref^158^.

In addition to using hydrogel-mediated mechanical cues for discovery, they are also valuable tools for tissue replacement. Hydrogels have been designed to promote desired cell morphologies, transduce applied forces, or relax to promote tissue regeneration. For example, PEG-hyaluronic acid hydrogels have been applied in concert with microfracture treatments in preclinical and clinical studies to treat cartilage defects^124^. The authors found that the hydrogels supported spherical cell morphologies, which promoted chondrocytic phenotypes and discouraged the typical fibroblast and osteogenic differentiation common in microfracture treatments. In another approach, hybrid scaffolds were made by 3D printing, followed by infilling with a modified PEG-based hydrogel to lend bioactivity (Figure 6A). These hydrogels were shown to restore chondral defects, in part by promoting retention of sulphated glycosaminoglycans in the tissue surrounding the defect^125^. Additionally, stress-relaxing poly(vinyl alcohol)-based hydrogels have been shown to support primary meniscal fibrochondrocyte culture and integration into excised meniscal tissue, highlighting the potential for stress-relaxation to drive the integration of implanted hydrogels in regenerative therapies^126^.

### Organoid development and disease models

Organoids provide an opportunity to study a simplified version of an organ in vitro, allowing researchers to better understand how mechanosensing impacts tissue-like structures^127^. Combining organoids with hydrogels has the potential to provide insight into how physical cues — including matrix elasticity and viscoelasticity — and applied forces impact organoid behaviour and are dysregulated in disease.

Different organoids are available, including intestine^128^, brain^129^, lung^130^, pancreas^131^, kidney^132^, and thymus^133^, many of which can be supported within synthetic hydrogels^134^. Use of synthetic PEG-based hydrogels to support murine intestinal stem cell (ISC) expansion and organoid formation was first described in REF^135^ (Figure 6B). ISC viability and expansion were maintained in nondegradable hydrogels with Young’s moduli of 1.3 kPa, which supported YAP nuclear localization. However, organoid formation, which is also dependent on continued YAP nuclear localization, was not sustained in these stiff matrices. Instead, hydrolytically degradable matrices were required to support ISC differentiation and organoid formation. It was later shown that the necessity for hydrolytic degradability could be supplanted by encapsulating intestinal organoids within synthetic hydrogels that allow for stress relaxation. By engineering reversible bonds into the system, mouse epithelial organoids could undergo crypt budding and human organoids could be supported long term^52^. Hydrogels that can be locally softened using photodegradation techniques and other bioengineering approaches have also been used to demonstrate how ISC activate differential notch signaling in response to local cell crowding induced by tissue curvature, triggering the differentiation of niche-supporting Paneth cells^136^.

Neural tube organoid growth has similarly been explored using hydrogels. For example, nondegradable hydrogels with an elastic modulus of 2 kPa were found to be optimal for neural tube organoid formation, as they prompted the formation of homogenous and polarized neuroepithelial colonies, as well as dorsal-ventral patterning reminiscent of neural tube architecture^137^. As the system is capable of mimicking key steps in early neurogenesis, it potentially enables exploration of neural signaling at early stages of development, with the opportunity to harness mechanical cues to direct these processes for regeneration. The earliest stages of development — epiblast formation by pluripotent stem cells — has also been studied using alginate hydrogels, with the finding that fast stress relaxation and high adhesion ligand density promotes the formation of lumen-containing structures that capture many features of the human epiblast^138^. More recently, hydrogels have been used to form arrays of round and pill-shaped microcavities that enabled standardized cultures of retinal^139^ and gut organoids^140^. Such systems are available commercially as Grid3D® (SUN Bioscience), allowing wide-spread adoption. Using these 2.5D systems, defined microwells, as well as hydrogels engineered with wave-like patterns, have allowed researchers to dissect the contributions of mechanical cues, including aspect ratio, stiffness and curvature, on the behaviour of cell monolayers and multicellular structures^141,142^.

In addition to fundamental mechanobiology studies, organoids also provide opportunities for tissue regeneration. For example, as Matrigel-supported organoid cultures are not suitable for translational applications, PEG-based hydrogels have been used to support human induced pluripotent stem cell (hiPSC)-derived intestinal organoid implantation in vivo to mediate repair^143^. This same hydrogel culture system was also successfully used to culture hiPSC-derived lung organoids, ultimately forming lumen and organized lung epithelium that are promising for lung repair applications^143^. In a complementary study, liver organoid formation was found to be sensitive to hydrogel stiffness, where YAP and Src family kinase activation were required for organoid formation^144^.

Hydrogels have also been used to explore the impact of mechanical cues in organoid-based disease models. For instance, hiPSC-derived kidney organoids cultured within soft, fast-relaxing alginate hydrogels were found to be more mature than those cultured in stiff hydrogels^19^. Remarkably, culture in stiffer or slower relaxing hydrogels also upregulated transcriptional markers associated with epithelial-to-mesenchymal transition, an early marker of renal fibrosis. Stiffer hydrogels resulted in decreased frequency and length of primary cilia, an ubiquitous cellular mechanosensor that has been implicated in fibrosis after acute kidney injury^145^. PEG-based hydrogels have been developed to support co-cultures of hiPSC-derived intestinal organoids with type 1 innate lymphoid cells (ILC1)^20^, which are known to accumulate in the inflamed intestines of patients with inflammatory bowel disease (IBD)^146^. IBD patients often develop fibrotic strictures, but the potential impact of ILC1 accumulation in the gut was previously unknown. The authors identified that ILC1 drove intestinal ECM remodeling, including increased deposition of fibronectin. These findings gave a potential link between ILC1 and intestinal fibrosis, made possible by the synthetic 3D culture system.

Such remodeling processes are also important in cardiac and other fibrotic diseases, where hydrogels provide opportunities for both fundamental and applied studies^147^. For example, tissue stiffening is associated with aortic valve stenosis and is part of a maladaptive feedback loop of myofibroblast activation and persistence^148^. PEG-based hydrogels with tunable 2D stiffnesses mimicking healthy and diseased cardiac tissue were used to identify a role for the mechanosensitive transient receptor potential vanilloid type 4 (TRPV4) calcium channel in myofibroblast activation. GelMA hydrogels have also been used for 3D co-culture of cardiac fibroblasts and cardiomyocytes for studying cardiac fibrosis. Whereas in 2D, cardiomyocytes become quiescent, in GelMA hydrogels, they formed a network capable of synchronous beating, establishing a model that can capture the transition from healthy to fibrotic cardiac tissue *in vitro*^149^.

Hydrogels have also been applied to study the tumour microenvironment in cancer, where physical cues are known to play important roles in tumour survival and metastasis^66,150,151^. For example, peptide-modified PEG-based hydrogels have been combined with pancreatic organoids to create ECM-specific tissue models. Softer hydrogels enabled the growth of healthy pancreatic ductal organoids (PDOs), while stiffer hydrogels could mimic the ECM of pancreatic ductal adenocarcinoma. Here, the authors found that PDOs responded to stiffer hydrogels with increased nuclear localization of YAP1, suggesting heightened mechanosignaling^18^.

### Immune cells and immunotherapy

Another emerging area where hydrogels are being applied is to understand and harness the impact of mechanical cues in immunology (Figure 6C). Immune cell responses to microenvironmental cues impact both innate and adaptive immune responses. Indeed, mechanical signals are crucial for immune cell migration, macrophage polarization, and T cell activation^6,152^. In particular, T cells sense and exert forces on their surrounding microenvironment^153^ and mechanosensing, which is essential for T cell receptor (TCR) complex-mediated activation, plays an important role in eliminating pathogens and cancer cells from the body^154^. Therefore, hydrogels have been harnessed to optimize T cell expansion, activation, and fitness^155,156^. For example, 2D polyacrylamide^157,158^ functionalized with immobilized antibodies has been used to promote Jurkat T cell proliferation and downstream signaling. In these studies, increased hydrogel stiffness in combination with immobilized anti-CD3 antibodies promoted IL-2 secretion^157,158^, a marker of T cell activation. Studies using 3D hydrogels have also provided additional insight into T cell activation and expansion, as they can mimic immune cell infiltration of 3D tissues^159^. For example, stiff alginate hydrogels promote increased T cell motility, higher proliferation, and enhanced activation and effector functions^160^. Presentation of T cell stimulating factors in 3D also significantly influenced T cell expansion, and cues conjugated to the matrix increased cell expansion compared to that observed in response to soluble cues alone. Taken together, these observations suggest a combined role for matrix stiffness, dimensionality, and ligand tethering in TCR-mediated T cell activation and response^161,162^.

T cell mechanosensing is also of importance for the manufacture of cell-based immunotherapies, such as chimeric antigen receptor (CAR)-T cell therapies for the treatment of haematological malignancies^163–165^. For example, hydrogels provide an opportunity to localize therapeutic cells to the desired tissue, retain cells long-term, and, importantly, stimulate cells for enhanced efficacy^164,166^. Indeed, CAR-T cells delivered within hyaluronic acid-based hydrogels showed enhanced T cell persistence compared to T cells delivered freely^167^. CAR-T cell-laden hydrogels have also been applied to eliminate tumour cells remaining after resection. The combined delivery of T cells and platelets, which release anti-PDL1 antibodies, prevented T cell exhaustion, ultimately decreasing tumour recurrence^167^. Additional work demonstrated that a chitosan-PEG hydrogel could simultaneously deliver CAR-T cells and IL-15 in a mouse model of retinoblastoma, eliminating retinoblastoma tumour cells without causing T cell-mediated vision loss^168^. Thus, the utility of hydrogels for the delivery of CAR-T cells has been established, and recent progress highlights the potential of further developing hydrogel-based mechano-immunostimulatory environments for enhancing the efficacy of therapies.

## Reproducibility and data deposition

Just as 2D cell experiments rely on tissue culture plastic manufacturers creating consistent and reliable plates and flasks, for hydrogels to stand on an equal footing, they similarly must be created and characterized consistently. Reproducibility in creating hydrogels is key to linking mechanical cues to biological outcomes. The challenge of creating reproducible hydrogels starts at the molecular level. For natural hydrogels, there can be significant batch-to-batch variation in the source material, which will impact the final hydrogel. This source of variability is inherently difficult to control. Therefore, it may be helpful to use the same large batch of material throughout an entire project.

Synthetic hydrogels do not suffer from the same inherent variability as natural hydrogels. However, their composition and properties can still vary. Because there are few commercial options for synthetic hydrogels, many laboratories manufacture their own. During a chemical synthesis, small changes in solvent composition, pH, salt concentration, and temperature can all have a large effect on the properties of the final hydrogel. Even seemingly minor issues, like the storing and handling of precursors, can impact the final product. Because of this, a good quality control (QC) system in which every batch is subjected to the same measurements from the molecular to macroscopic level is important. Relevant approaches for ensuring consistency in hydrogel properties include spectroscopic, chromatographic, and biochemical techniques to characterize the material’s functionality, molecular weight, and concentration. For example, NMR can be used to examine the number and type of functional groups per macromolecular building block. Liquid chromatography can be applied in tandem with mass spectrometry or light scattering to examine molecular weight and functionality. Similarly, biochemical assays, such as Ellman’s assay, which quantifies the free thiols that are crucial for forming hydrogels using a Michael addition, are often necessary to ensure consistency. UV-vis spectroscopy can also be used to assess functional group or building block concentration in precursor solutions^80^.

Once QCs are established for synthesis, systematic testing of hydrogel properties ensures consistency of experimental results. For example, shear rheometry or other mechanical testing techniques and equilibrium swelling assays should be used to assess the rate of hydrogel formation and resulting mechanical properties and cross-link density. Bioactivity should also be tested to ensure batch quality. For example, RGD-containing or other adhesive peptides tethered to synthetic hydrogels can be tested by assessing cell attachment on the 2D surface. It is of particular importance to use standardized cell lines and protocols for functional QCs. Similarly, ECM-based hydrogels that are intended to support organoids can be tested by assaying organoid phenotype. Standard operating procedures, which include such QCs, can ensure hydrogel reproducibility. With this approach, inherent biological variability due to donor, cell heterogeneity, or other factors are not further confounded by materials issues.

Beyond QC, sources of error within an experiment or measurement need to be carefully considered and controlled to ensure reproducibility. For example, to account for error in weighing and pipetting when assessing mechanical properties, three different precursor stock solutions can be prepared — each from dry powder — and the rheological properties of at least two hydrogel replicates measured from each stock solution. To account for spatial heterogeneity and provide sufficient sampling, AFM measurements should be performed by probing the material multiple times at random locations. For example, to assess modulus, measurements could be performed across 10 × 10 grids in 100 μm × 100 μm maps, at six locations on the surface to produce 600 force-displacement curves ^86^. In all cases, the resolution of the instrument should be considered — such as the minimum mass, volume, concentration and modulus — and measurements performed sufficiently above the detection limit. When assessing biological responses, replication within the experiment and across multiple experiments must be considered to account for sources of variability and allow appropriate statistical analyses.

As has become standard in the biological sciences, open depositing of data should be adhered to when working with cells in hydrogels. Placing large datasets in repositories with appropriate metadata and linking them to one another, for example simultaneous RNAseq and proteomics, provides a shared data mining source for the community. Additionally, such resources allow researchers to reuse and compare data between labs and hydrogel systems. As there are no recognized guidelines, an opportunity exists within the hydrogels for mechanobiology community to begin establishing such repositories and standards, building from successful frameworks that have been developed with the data science and molecular biology communities. It is suggested to follow existing standards and place gene expression data in the Gene Expression Omnibus^169^ or the Annotare database^170^; proteomics data in the ProteomeExchange^171^; and code in repositories such as GitHub. It is also important to create standards for reporting characteristics of the material itself. Box 2 details recommendations for the minimum information that should be reported for any hydrogel experiment, which is intended to act as a standard.

## Limitations and optimizations

The simplicity of many engineered hydrogels as ECM biomimetics presents both opportunities and limitations. On one hand, this simplicity can provide model system for reductionist research aimed at uncovering basic mechanisms of cell-ECM interplay^172,173^ and how mechanical forces drive these processes. On the other hand, their lack of biological complexity and the limited range over which their mechanical properties can be modulated constrains the scope of applications in which hydrogels can be applied^174^. The quest for minimal hydrogel complexity that can achieve robust biological outcomes is perhaps the major driving force for innovation in both fundamental discovery and biomedical application. However, the accessible range of hydrogel properties is ever increasing, and innovations continue to push the limits of achievable mechanical strengths^175,176^, viscoelastic characteristics^84^, molecular-scale biofidelity^177^, and multi-scale architectures^178^. The field is also making important advances in controlling temporal evolution of biophysical and biochemical hydrogel properties^179^, including creating predictable cell-driven evolution across days, weeks, and even months.

At present, biologically derived and synthetic hydrogels generally lack supracellular tissue structures such as nerves, blood vessels, and ECM fibers with long-range order. This limits the cellular responses that may be studied in vitro, for instance in the context of cancer where these structures play important roles in mechanobiology-driven processes like metastasis^180^. Hydrogels are often homogenous and the techniques used to characterize them focus on examining their bulk properties. However, cells within tissues experience gradients and abrupt changes in local mechanical properties^181^ across a range of scales. Fibrous hydrogels, which incorporate fragmented fiber mats created using electrospinning techniques^182^, as well microgels, or hydrogel microparticles processed and manipulated at the micron scale, may address some of these concerns as they provide this longer-range order and allow for local variations in mechanical or biological properties^183,184^, respectively. Nevertheless, even when combined with high-resolution bioprinting, they still remain far from truly replicating the complexity of native tissue architectures.

The fidelity of synthetic hydrogel biomimicry is also confined by the ability to impose sufficiently complex yet controlled biological cues. Although hydrogels can be engineered to provide well-defined physical properties at the subcellular scale, approaches to implement biological cues at this scale remain limited. Minimal peptides that are used for cell binding do not fully replicate biological interactions with native matrisome proteins. Chemistry techniques that have been harnessed to incorporate full proteins often change protein function in unknown ways that may limit their utility. Biotechnology approaches to expand the range of embeddable biological cues^185^ have their own shortcomings, such as reliance on bacterial expression. In any case, use of biologically derived materials, whether consisting of bacterially expressed matrisome fragments or a native matricellular sugar like hyaluronic acid, can lead to uncontrolled biological interactions. While these interactions may be helpful to achieve some targeted biological outcomes, they can also confound fundamental studies aimed at understanding how mechanical cues impact cellular responses.

The challenge in predicting cell-ligand interaction is substantially deepened by the fact that cells are not passive sensors within 3D hydrogels. Cells continuously secrete and arrange proteins around themselves. The paradigm that cells will sense and react to hydrogel cues and the expectation these cues will continue to instruct embedded cells warrants critical assessment^123^. Beyond pushing the limits of achievable biophysical and biochemical hydrogel biomimicry, improved biological tools for the study of mechanobiology are needed. Existing mechanobiology tools generally focus on the cell, with biological activity reporters that span the cytoskeleton, gene transcription, epigenetic reading and writing machinery, protein phosphorylation, mitochondrial metabolism and beyond. In stark contrast, the range of established and available extracellular reporters is very limited. Some notable progress has been made including FRET-based sensing of matrix tension^186^ and displacement-based traction force microscopy approaches^187^. However, these tools are not easily accessible and are difficult to interpret. Expanding the range of matrix reporters will be essential to make real progress in biomimetic hydrogels, particularly in tools that can quantify the cell-level stimuli that elicit mechanotransduction responses. The required range of reporters extends to the biochemical composition of the pericellular matrix itself which can play an important role in context-dependent mechanotransduction^188^.

## Outlook

Hydrogels have allowed researchers to explore the impact of isolated mechanical and biological cues on cellular responses. However, as the field develops, many simple hydrogels are likely to be replaced with systems that feature increased, but controlled complexity. Many researchers still rely on ECM-based or synthetic hydrogels containing simple ECM-mimicking peptides. However, in the future, incorporation of more complex and tissue-specific matrix cues will enable researchers to study how mechanosensing is altered by specific matrisome interactions. It is also foreseen that additional architectural, biological and physical cues will be incorporated into hydrogels. These might include electrical stimulation or interstitial flow, as well as functional vasculature, nerves and different immune cell populations. As understanding of the importance of viscoelastic cues in biological response grows, the field will need to develop hydrogels that can better match the quick responses reported in native tissue that are often on the order of seconds^64^.

It will be important for many disease modelling applications to mimic the long-range fibrous structure of the native ECM in engineered models. This will be key to developing advanced cancer models, where tumour cells use blood vessels, nerves and matrix fibers to move collectively^4^. Efforts to incorporate fibrous structures formed using electrospinning techniques have been reported^182,189^. Here, encapsulated cells proved capable of recruiting fibers, creating local fiber concentration gradients, and aligning in the direction of cell-generated stresses, reminiscent of cell behaviours when encapsulated in collagen hydrogels. Future studies should look to demonstrate the utility of these models in replicating in vivo-like cellular behaviours, such as those observed in animal models using intravital microscopy^190,191^. In short, by combining hydrogels with more advanced matrisome cues, fibrous assemblies, organ-on-chip technologies^192^ and bio-printing approaches^193^ capable of providing architectural complexity, it may be possible to create multi-component tissue models suitable for discovery and drug screening.

Both during development^181^ and in pathological processes such as fibrosis in the lung and liver, the mechanical properties and composition of the ECM change with time. As researchers aim to study these processes in vitro, it will be become increasingly important to create hydrogels with user-controlled means to match these cues. For example, hydrogels that can controllably soften upon exposure to UV light are well documented^28^ and have recently been applied to organoid cultures^136^. Similarly, secondary cross-linking strategies^14^ that stiffen the hydrogel and chemistries that allow for cyclic softening and stiffening^194^ have been reported. However, hydrogels that allow for the real-time modulation of matrisome cues are less advanced. For example, photo-mediated methods have been applied to remove RGD sequence containing-peptides from cell-laden hydrogels, resulting in reduced cell viability^28^, and reversible bioconjugation strategies have been applied to 3D hydrogels to pattern and release thiolated proteins^195^. These approaches allow for only limited control of proteins or peptides with specific features. An enzyme-triggered transpeptidation strategy that installs a handle for tethering a protein into a hydrogel using a photo-mediated ligation technique was recently described that would enable more complex patterning of molecules in 3D hydrogels^185^. However, even this strategy remains far from replicating the dynamism and complexity of the native matrisome, particularly as the modulation of matrisome cues on demand during culture is limited to proteins that can be expressed recombinantly. Additionally, their incorporation into 3D hydrogels relies on diffusion, which can be an issue for large proteins in stiff hydrogels.

The emergence of -omics technologies has revolutionized biology and they are being progressively applied to hydrogel cultures. However, as these approaches increasingly rely on intact single cells, future hydrogel designs will require quick, but gentle and robust strategies to release single cells and organoids without impacting biological readouts. For covalently cross-linked networks, chemistries that rely on non-mammalian enzymatic approaches, such as Sortase A^196,197^, or temperature-induced disruption^32^ enable rapid recovery of single cells and multicellular constructs. This allows for re-encapsulation or downstream analysis, including using emerging approaches in metabolomics, lipidomics and metallomics. To harness the power of such techniques, these and similar hydrogel chemistries will need to be adopted.

Spatial transcriptomic and mass spectrometry techniques have the potential to provide unprecedented insight into how single cells, particularly in multi-cellular structures such as organoids, respond to mechanical cues. Thus far, these techniques have not been applied extensively to hydrogel cultures. While FRET-based approaches are increasingly being used as intracellular mechanosensors, the hydrogel community still relies on crude techniques, such as traction force microscopy and microrheology, to quantify matrix remodelling in the extracellular space. The wide-spread application of advances, including the incorporation of FRET-based sensors into hydrogels to read out cell-mediated tension, would enable more precise measurements of mechanically driven processes and cellular interactions with their surrounding matrix.

Designing new hydrogel chemistries to meet emerging challenges in biology remains an ongoing challenge. Early approaches were often designed and tested on robust mesenchymal cells and transformed cell lines. However, as the applications of hydrogels expand, the community needs new gelation strategies that avoid damaging UV light and potentially cytotoxic cross-linking chemistries. The growing interest in organoid-based disease modelling demands hydrogels that are very soft, but that can cross-link quickly. Applying molecular dynamics simulations or machine learning approaches to predict cross-linking of novel designs has the potential to identify chemical strategies that maximize cross-linking kinetics while minimizing stiffness. Finally, as researchers move into culturing in 3D, where stiffness, viscoelasticity, degradability and ligand density, among other factors, can all be modulated, the potential experimental landscape grows exponentially. With so many experimental options to explore, taking a purely empirical approach can be daunting. However, techniques in mathematical modelling are increasingly being applied. For example, an in silico digital twin^198^, can be used to generate and test hypotheses, interrogate the system’s sensitivity to various experimental factors, and inform experimental decisions. Such approaches have the potential to reduce the number of factors that must be tested experimentally.

As researchers continue to develop hydrogels that can better replicate the multi-scale biological and mechanical complexity of the native ECM, the fidelity with which engineered tissue created in vitro will match those of native tissues will increase. Combining these next generation hydrogels with multi-omic-based knowledge of biological systems has the potential to lead to new mechanistic understanding into how mechanical cues impact normal physiology and disease. Such insights have the potential to rival those produced by the molecular revolution which has dominated biomedicine over the past decades, and allow for tissue engineering’s promise of replacement tissues and organs to be realized.

## Figures and Tables

**Table 1:** Challenges in applying hydrogels in mechanobiology studies.

CHALLENGE	CHALLENGE TYPE	PRESENT LIMITATIONS	FUTURE SOLUTIONS	PROPOSED INTERIM WORKAROUNDS
**Hydrogel biomimicry of the ECM**	**Technical**	Limited control over cell-scale architecture. Limited capability to produce supracellular architectures. Limited capability for multi-scale manufacture of tissue-like structures.	Next generation biofabrication approaches that enable scalable production of defined supramolecular architectures, including microporosity and large aspect ratio fibrous structures. Composite materials that capture biological features of fibers and interstitial spaces.	Hydrogel chemistries that rely on nano/microscale cues that robustly guide short-term in vitro cellular behaviours and remodeling of the pericellular environment. Hydrogels with tissue-level cues that accurately reflect pericellular and supracellular in vivo niches.
	**Biological**	Ligand complexity insufficiently reproduced. Ligand mobility and tethering do not accurately replicate native interactions. Non-physiological spatio-temporal ligand distributions and gradients.	Technologies combining fiber assembly with chemical approaches to permit fuller control of ligand complexity and spatial distribution. Reductionist cellular niches to deterministically guide cell behavior.	Approaches that provide limited yet robust subsets of biophysical/biochemical cues. Systematic efforts to validate *in vitro* outcomes against *in vivo* experiments by benchmarking against multi-omic analyses of human cells and tissues.
**Cell-level biophysical measurements**	**Technical**	Intractable force microscopy setups and datasets. Non-standardized biophysical readouts. Overly simplified load cases. Over-weighted focus on cells rather than matrix.	Improved experimental tools including expanded range of microscopy-based approaches to analyze cells and *matrix*. High-throughput single- and multi-cell micro-engineered assays with controlled biophysical and biochemical boundary conditions.	Lower-resolution approaches (temporally, spatially, biologically) that serve as rough proxies of relevant cell and tissue behaviours.
	**Biological**	Relevance of single cell biophysical behaviors at short timescales. Simplified cell models that fail to replicate in vivo cellular and tissue complexity.	Biologically relevant, tissue specific, biophysical outcome metrics that are robustly related to multi-omic cell/tissue signatures in health and disease.	Correlation of lower resolution readouts to known disease phenotypes, such as metastatic invasiveness, hypercontractility in fibrosis, and mucoid degeneration.
**Overarching knowledge gaps**	**Biological**	Understanding of the cellular niche, including tissue-specific homeostasis, and cell/tissue response to damage and disease, and during regeneration. Robust understanding of cellular mechanosensors and the context-dependent pathways they act through.	Whole body single cell transcriptomic/proteomic/metabolomic atlases with integrated understanding of single- and multi-cellular behaviors in the context of health and disease.	Increasing insight into single and multi-cell behaviors supported by increasingly broad and deep multi-omic datasets generated using best practices for findable, accessible, interoperable, and reproducible science.

## Box 1 Strengths and limitations of natural and synthetic hydrogels

### Natural hydrogels

#### Strengths

- Long history of straightforward use within the biology community.
- Inherent bioactivity that enables cell viability and cell adhesion.
- Proteolytically degradable and amenable to cell-mediated remodeling

#### Limitations

- Batch-to-batch variability, depending on tissue source and manufacturing process.
- Low mechanical stiffness, which is often limited by protein solubility and physical cross-linking methods. Matrigel’s elastic modulus is ~ 100s Pa.
- Mechanical properties can only be tuned over a limited range.
- Biological and mechanical cues often cannot be modified independently.
- Contain confounding biological factors, such as growth factors.

### Synthetic hydrogels

#### Strengths

- Blank slate that is free of confounding proteins and animal products.
- Mechanical properties can be tuned independently of biological cues.
- Mechanical properties can be varied widely to create materials with elastic moduli in the range of ˜ 100s Pa to 100s kPa.
- High reproducibility due to the defined chemical synthesis of the material.

#### Limitations

- In house chemistry techniques required to modify polymers are not available within biology laboratories.
- Require chemical addition of biological cues to render them bioactive.
- Do not capture full biological/structural complexity of the native extracellular matrix.
- Few commercial options.

## Box 2 Recommended hydrogel parameters that should be reported for reproducibility

### Hydrogel building blocks

- Polymer or biopolymer identity, source, and manufacturer or vendor.
- Polymer or biopolymer molecular weight.
- Polymer or biopolymer functional group identity and degree of modification.
- Initiator or catalyst identity, if applicable.
- Identity of other monomers, such as peptide linkers, cell-interactive groups, or ligands, including manufacturer or vendor information, if applicable.
- Methods for synthesis or modifications of materials made in house.

### Hydrogel formation

- Monomer stock solution concentrations.
- Buffer information, including salt concentrations and pH if not a defined or standard.
- Final concentrations of all components in the hydrogel, including polymer, peptides and proteins.
- Mixing methods, particularly with cells or assembled proteins and peptides for consistency.
- Hydrogel volume and geometry, including any information on mould or if formed in situ.
- Hydrogel crosslinking chemistry.
- Specifications of any external triggers, for example light wavelength, intensity, exposure time.
- Gelation conditions, such as time, temperature and pH.

### Cell incorporation, if applicable

- Cell type(s) and source.
- Procedures for cell isolation, modification, and/or differentiation, if applicable.
- Cell expansion methods and reagents.
- Passage number at seeding or encapsulation.
- Cell seeding density (2D or 2.5D hydrogels) or encapsulation density (3D).
- Cell culture reagents and conditions.
- Specifics for handling procedures that deviate from typical sterile technique.
- Methods for cell characterization at various stages in workflow and assessment of responses.

### Characterization of hydrogel properties

- Mechanical properties, such as elasticity and viscoelasticity.
- Crosslink density and mesh size.
- Swelling behavior, for example swelling ratio.
- Degradability (degradation kinetics in response to internal or external stimuli)

## Glossary terms

### Abbreviations

Anoikis: Cell death in anchorage-dependent cells driven by a lack of cell-ECM interactions.
Creep: The process by which a viscoelastic material undergoes gradually increasing and irreversible deformation in response to a constant applied force.
Disease modeling: The modeling of a diseased tissue through animal models or tissue-engineered scaffolds to study disease progression and possible treatment.
Elastic modulus: The tangent of a material’s stress-strain curve, which is an intrinsic property of the material. Modulus is a measure of stiffness that is independent of the material’s size and geometry.
Matrix metalloproteinase: A class of cellular proteases that biochemically degrade extracellular matrix proteins.
Mechanosensing: The ability of a cell to probe or sense mechanical cues of its extracellular environment, including force, stress, strain, confinement, and substrate topology.
Mechanotransduction: The cellular conversion of a mechanical stimuli into biochemical signaling, such as via activation of receptors, signaling pathways, transcriptional activity, or protein activation.
Mesh size: The distance between polymers or crosslink points in a polymer network. Mesh size can impact solute diffusivity.
Microrheology: A microscale measurements of the mechanical properties of a material.
Organoid: A three-dimensional stem cell-derived tissue culture that exhibits aspects or properties of the parent organ.
Stiffness: A material’s ability to resist deformation in response to an applied force.
Stress relaxation: The process by which a viscoelastic material, which initially resists an applied strain, reduces its resistance to the deformation over time. In hydrogels this often related to fluid movements within the solid material backbone, or, in weakly crosslinked hydrogels, unbinding of weak crosslinks followed by polymer flow.
Stress stiffening: The process by which materials stiffen due to an applied strain, sometimes as a result of fiber alignment.
Tissue engineering: The combination of biologically active materials platforms with cells to develop systems that may be used to restore or treat damaged tissues.
Viscoelasticity: A mechanical response on a material that combines characteristics of viscous liquids and elastic solids.
